# Accessing the Impacts of the Calculated Panel Reactive Antibody Value on a Lung Transplant Waitlist: A Latin American Experience

**DOI:** 10.3390/jcm14124344

**Published:** 2025-06-18

**Authors:** Samuel Lucas dos Santos, Flavio Pola dos Reis, Luis Gustavo Abdalla, Lucas Matos Fernandes, Elissa Ayumi Okuno, Priscila Cilene Leon Bueno Camargo, Rafael Medeiros Carraro, Silvia Vidal Campos, Ricardo Henrique Oliveira Braga Teixeira, Paulo Manuel Pêgo-Fernandes

**Affiliations:** Instituto do Coração, Hospital das Clinicas, Faculdade de Medicina, Universidade de Sao Paulo, Sao Paulo 04023-900, SP, Brazil; flavio_pola@hotmail.com (F.P.d.R.); elissa.okuno@hc.fm.usp.br (E.A.O.); silvinhavc@uol.com.br (S.V.C.); rhobteixeira@terra.com.br (R.H.O.B.T.);

**Keywords:** lung transplant, lung transplantation, panel-reactive antibody, transplant waitlist

## Abstract

**Backgorund\Objectives:** Lung transplantation is the definitive treatment for select patients with end-stage pulmonary diseases. However, immunologic sensitization, as measured by calculated panel-reactive antibody (cPRA), poses significant challenges to transplant access and outcomes. This study aimed to evaluate the impact of cPRA on lung transplantation waitlist dynamics in a single-center cohort in Brazil, focusing on its association with waitlist mortality, delisting, and transplantation. **Methods:** A retrospective cohort study was conducted including all lung transplant candidates listed in our institution between January 2012 and December 2022. Candidates were stratified by cPRA values at listing into five groups: 0%, 0.1–25%, 25.1–50%, 50.1–75%, and 75.1–100%. Primary outcomes included lung transplantation, with secondary outcomes of waitlist mortality and delisting due to clinical deterioration. Statistical comparisons were performed, as appropriate. **Results:** Of the 411 candidates evaluated, 327 met the inclusion criteria. Among them, 100 (30.6%) were sensitized (cPRA > 0%), with increasing cPRA values correlating with longer median waitlist times (*p* < 0.01). Although transplantation rates were not statistically different across the cPRA strata (*p* = 0.277), the group with a cPRA > 75% had the lowest transplant rate (37.5%). Waitlist mortality was significantly higher in candidates with a cPRA > 50% (*p* = 0.047), whereas delisting rates did not differ across groups (*p* = 0.722). **Conclusions:** Elevated cPRA is associated with prolonged waitlist time and increased mortality, reflecting both immunologic and logistical barriers to lung transplantation. These findings support the need for incorporating cPRA into allocation policies and adopting targeted strategies, such as desensitization protocols, to improve equity in transplant access for sensitized patients, particularly in genetically diverse populations. Further multicenter studies are warranted to validate these results and inform policy development.

## 1. Introduction

Lung transplantation remains the definitive therapeutic option for selected patients with end-stage pulmonary diseases. However, its long-term success is often undermined by immunologic complications, particularly those mediated by mismatches in the human leukocyte antigen (HLA) system [1,2,3]. The HLA complex molecules are expressed on the surface of donor cells and are the primary targets of the recipient’s immune response [4]. Incompatibilities in HLA matching have been strongly associated with the development of donor-specific antibodies (DSAs), acute cellular rejection, and chronic lung allograft dysfunction (CLAD), ultimately compromising graft and patient survival [5].

Beyond the complexity of HLA compatibility, allosensitization has emerged as a critical barrier to lung transplantation. Allosensitized patients possess pre-formed anti-HLA antibodies, which significantly reduce the pool of acceptable donors [6]. Calculated panel-reactive antibody (cPRA) is a validated tool used to quantify the proportion of donors that are incompatible with a given candidate based on their HLA antibody profile. A higher cPRA reflects a broader sensitization, indicating that a patient has antibodies against a larger percentage of potential donors [7].

Studies have shown that lung transplant candidates with elevated cPRA levels experience prolonged waitlist times, reduced likelihood of receiving a transplant, and increased risk of death while waiting [3,5,7]. However, once transplanted, survival outcomes between sensitized and non-sensitized patients appear comparable, emphasizing that the primary burden of sensitization lies in access to transplantation rather than post-transplant prognosis [8,9].

When a suitable donor becomes available, histocompatibility laboratories perform virtual crossmatching and antibody verification using high-sensitivity methods. These technologies allow precise identification of donor-specific HLA antibodies, enhancing the safety of donor selection for sensitized recipients [6]. Nevertheless, the complexity of interpreting antibody strength, complement-binding capacity, and clinical relevance requires a nuanced approach to donor acceptance in sensitized patients.

Additionally, the impact of HLA variability is shaped by population-level genetic diversity. In genetically homogeneous populations, the likelihood of identifying compatible donors is higher, reducing the consequences of sensitization. In contrast, in heterogeneous or admixed populations, as evidenced in larger Latin American countries, broader HLA variability can significantly limit donor options for sensitized individuals, exacerbating disparities in transplant access [10]. This underscores the importance of tailoring allocation strategies and immunologic risk assessment tools to population-specific contexts.

Given these multifaceted challenges, understanding the impact of cPRA on lung transplant waitlist dynamics is essential for informing equitable allocation policies and improving outcomes for sensitized patients. This study aims to evaluate the role of cPRA in influencing lung transplantation access in a single-center cohort, with a focus on its relationship to waitlist mortality, delisting, and eventual transplantation.

## 2. Materials and Methods

A retrospective study was conducted at a single lung transplantation center in Brazil—the Instituto do Coração, Hospital das Clínicas, Faculdade de Medicina, Universidade de São Paulo. The study utilized a database encompassing all lung transplant candidates listed at the institution between 1 January 2012, and 31 December 2022. Data were extracted from electronic medical records and the REDCap (Research Electronic Data Capture – version 14.5.21) platform at our institution. The variables collected included gender, age, blood type (A, B, AB, and O), body mass index (BMI), and primary lung disease.

All candidates underwent panel-reactive antibody (PRA) testing at the time of listing. Only cPRA values for class I and class II HLA were included in the analysis. Currently, the identification of anti-HLA antibodies is primarily performed using assays based on single antigen beads (SABs), which enable the detection and characterization of antibodies present in the recipient’s serum against HLA antigens from various loci (typically HLA-A, -B, -C, -DRB1, -DQB1, and potentially including DP and others). It is important to highlight that both the methodology and the accuracy of these techniques have significantly improved over the final years of this study. The evaluation of potential donors was carried out according to standard procedures, and all recipients initially underwent virtual crossmatching. Crossmatches were considered negative in the absence of anti-HLA antibodies and positive when such antibodies were detected.

Patients who underwent retransplantation, those delisted for reasons unrelated to clinical deterioration (e.g., transplant refusal or diagnosis of neoplasm), and patients transplanted under prioritization protocols were excluded from the analysis.

The initial comparison was made between two groups: candidates with negative classes I and II cPRA and those with positive classes I and/or II cPRA. Subsequently, candidates were stratified into five groups based on cPRA values at the time of listing: 0%, 0.1–25%, 25.1–50%, 50.1–75%, and 75.1–100%, considering either HLA class.

In both analyses, the primary outcome was receipt of lung transplantation, while secondary outcomes included delisting due to clinical deterioration and death while on the transplant waiting list.

Baseline characteristics of the cohort were summarized using medians with ranges or interquartile ranges (IQRs) for continuous variables and frequencies with percentages for categorical variables. Continuous variables were compared using the *t*-test or analysis of variance (ANOVA), as appropriate. Categorical variables were assessed using the chi-square test. Statistical analyses were performed using IBM SPSS Statistics version 30.0 (Armonk, NY, USA), with a significance level set at *p *< 0.05.

## 3. Results

A total of 411 candidates were listed for lung transplantation between 1 January 2012 and 31 December 2022. Of these, 84 candidates were excluded from the analysis: 31 due to missing cPRA values; 2 due to a diagnosis of neoplasm while on the waiting list (1 with thyroid cancer and 1 with lung cancer); 2 who declined lung transplantation; 10 who underwent lung retransplantation; and 39 who received transplants under a prioritization protocol.

**Figure 1:**
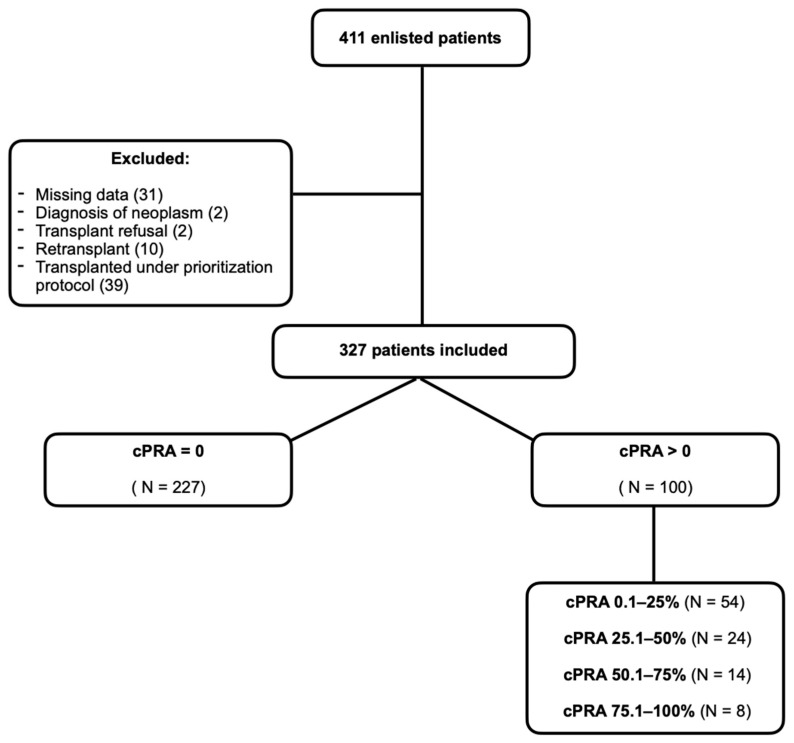
Distribution of the included candidates for lung transplant on waiting list from 1 January 2012 to 31 December 2022 according to cPRA value and stratification.

The final study cohort comprised 327 candidates. Among them, 227 (69.4%) had a class I and/or class II cPRA of 0%, while 100 (30.6%) were considered sensitized, presenting with cPRA values greater than 0%. Within the sensitized group, 54 candidates (54.0%) had a cPRA between 0.1 and 25%, 24 (24.0%) between 25.1 and 50%, 14 (14.0%) between 50.1 and 75%, and 8 (8.0%) between 75.1 and 100%. This distribution is illustrated in Figure 1.

The included candidates had a median age of 41 years (range: 10–67 years). When comparing non-sensitized individuals (cPRA = 0%) to sensitized individuals (cPRA > 0%), no statistically significant difference in age was observed (*p* = 0.958). The majority of patients in both groups were female—66% in the sensitized group and 52.4% in the non-sensitized group—with a statistically significant difference in gender distribution (*p* < 0.01).

Regarding blood group distribution, a higher proportion of sensitized candidates belonged to blood group A (49%) compared to those in the non-sensitized group (39.6%), a difference that was also statistically significant (*p* < 0.01). These demographic and baseline characteristics are detailed in Table 1.

Of the 327 candidates included in the analysis, 230 (70.4%) underwent lung transplantation, 72 (22.0%) died while on the waiting list, and 25 (7.6%) were delisted due to clinical deterioration, becoming ineligible for transplantation. Notably, mortality while on the waiting list was significantly higher in the sensitized group compared to the non-sensitized group (27.0% vs. 19.7%, *p* < 0.01).

The demographic and clinical characteristics of the transplanted patients are summarized in Table 2. The median age at the time of listing was 44 years (range: 10–66 years), with a predominance of female recipients (53%). The most common blood type was group A, observed in 108 patients (46.9%).

Among the 230 patients who underwent lung transplantation, the most frequent underlying diagnosis was interstitial lung disease (n = 98; 42.6%), followed by cystic fibrosis (n = 54; 23.5%), obstructive lung disease (n = 37; 16.1%), non-cystic fibrosis bronchiectasis (n = 33; 14.3%), and pulmonary vascular disease (n = 8; 3.5%).

The median BMI at the time of listing was 22.3 kg/m^2^. The median duration on the waiting list was 551 days (range: 35–3151 days). With respect to sensitization status at the time of listing, class I cPRA values ranged from 0% to 75%, while class II cPRA values ranged from 0% to 99%.

**Figure 2:**
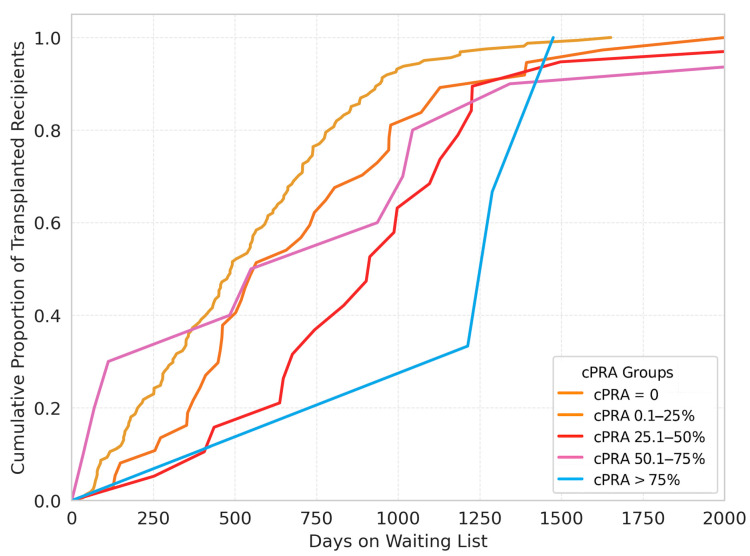
Proportion of transplanted patients according to the stratification by cPRA range value.

In the stratified analysis of the 327 patients included in this study, detailed in Table 3, the overall lung transplantation rate was 70.9% in the non-sensitized group (cPRA = 0%). Among sensitized patients, transplantation occurred in 37 individuals in the cPRA 0.1–25% group, 19 in the 25.1–50% group, 10 in the 50.1–75% group, and 3 in the >75% group. This distribution did not reach statistical significance (*p* = 0.277). The cumulative proportion of the transplanted patients according to the time on the waitlist is presented in Figure 2.

The median waiting list times increased progressively with higher cPRA values, with medians of 489, 565, 913, 742.5, and 1288 days for the cPRA = 0%, 0.1–25%, 25.1–50%, 50.1–75%, and >75% groups, respectively, demonstrating a statistically significant difference across groups (*p* < 0.01).

Mortality while on the transplant waiting list was proportionally higher among patients with a cPRA > 50%, with a statistically significant association observed (*p* = 0.047). In contrast, no significant difference was found in the distribution of delisted patients across the groups (*p* = 0.722).

## 4. Discussion

The primary objective of this study was to evaluate the impact of cPRA on access to lung transplantation within our institutional cohort, with particular emphasis on its association with waitlist mortality, delisting, and receipt of transplantation. The findings support the utility of cPRA as a relevant immunologic metric influencing transplant accessibility.

Although the overall transplantation rate did not differ significantly across the cPRA-stratified groups in our study, the trend toward lower transplantation rates in highly sensitized patients is consistent with findings from previous literature on the topic. Aversa et al. [11], for instance, demonstrated that lung transplant candidates with cPRA values between 50.1 and 75% were 25% less likely to undergo transplantation, while those with cPRA values between 75.1 and 100% experienced a 52% reduction in transplant likelihood. In our cohort, the group with a cPRA > 75% exhibited a markedly lower transplantation rate (37.5%) compared to the other groups, aligning with these prior observations. The absence of statistical significance in our analysis (*p* = 0.277) may be attributed to the limited sample size, particularly within the most highly sensitized subgroups, which reduces the power to detect meaningful differences. Nevertheless, the observed trends suggest that cPRA remains a critical factor in lung transplant accessibility, and future studies with larger, multicenter populations are warranted to further elucidate these associations.

The progressive increase in median waiting time with higher cPRA values highlights one of the most significant clinical consequences of allosensitization. As patients with elevated cPRA must wait longer for a compatible organ, they are at greater risk of deterioration and mortality, particularly in healthcare systems with limited donor availability. The association between increasing cPRA values and higher waitlist mortality is supported both by the findings of the present study and by previously published literature on the topic. In our cohort, a greater proportion of deaths was observed among candidates with higher cPRA values, particularly those exceeding 50%, reinforcing the negative impact of allosensitization on waitlist outcomes. This observation is consistent with the study by Tague et al. [6], which demonstrated a statistically significant association between rising cPRA levels and the risk of death on the waiting list. Specifically, each 10% increase in cPRA was associated with a 15% increase in the subhazard for waitlist mortality (sHR = 1.15; 95% CI, 1.07–1.22; *p* < 0.001). Similarly, Barac et al. [7] reported that candidates with a cPRA ≥ 50% had a significantly elevated risk of waitlist mortality at one year, with a hazard ratio of 1.71 (95% CI, 1.55–1.88; *p* < 0.001).

Delisting due to clinical deterioration is a complex, multidisciplinary decision that typically involves the integration of clinical judgment, objective assessments, and institutional protocols. In our study, the rate of delisting did not significantly differ across the various cPRA strata, a finding inconsistent with previous reports [11]. This suggests that while higher cPRA levels are clearly associated with increased waitlist mortality, they may not necessarily correspond with clinical deterioration severe enough to meet delisting criteria. Alternatively, this finding may highlight heterogeneity in the criteria used for delisting across time and across different institutions. The absence of standardized guidelines means that delisting decisions are often guided by institution-specific thresholds, incorporating both qualitative and quantitative clinical and immunologic parameters, which may vary considerably between centers and evolve over time.

In light of the challenges associated with delisting sensitized patients, recent studies have proposed innovative strategies aimed at improving transplant opportunities through the reduction in cPRA. Comins-Boo et al. and Castro Hernández et al. [12,13] have described approaches to lower cPRA values via structured, low-risk delisting protocols in kidney transplant candidates. These strategies include reassessment using single-antigen bead assays with diluted serum and redefinition of unacceptable antigens based on historical mean fluorescence intensity (MFI) values, with the goal of reducing cPRA below 99.95% to increase the probability of receiving a donor offer. In the study by Comins-Boo et al. [12], such approaches successfully resulted in cPRA reduction and increased transplantation rates among highly sensitized patients. While these strategies have been developed primarily in the context of kidney transplantation, their underlying principles may be applicable to lung transplant candidates as well. Implementing similar delisting and reassessment protocols could offer an opportunity to optimize donor compatibility and enhance access to transplantation in sensitized individuals. Further research is warranted to explore the efficacy and safety of such approaches in lung transplantation settings, particularly given the unique immunologic and clinical challenges faced by this population.

Genetically diverse populations, where the variability in human leukocyte antigen (HLA) types can complicate matching and increase sensitization rates. In admixed populations such as Brazil, transplant programs should consider adopting strategies aimed at improving access for these high-risk groups. These strategies may include desensitization protocols, prioritization algorithms based on cPRA, and investment in virtual crossmatch capabilities.

The medical literature on the topic outlines several approaches to desensitization. One common strategy involves perioperative desensitization protocols that include plasma exchange, intravenous immune globulin (IVIG), and antithymocyte globulin. This approach has been shown to allow safe transplantation in donor-specific antibody (DSA)-positive patients, with outcomes comparable to unsensitized recipients [8,14]. Another plausible approach is the use of a multi-modal desensitization protocol, which may include plasmapheresis, solumedrol, bortezomib, and rituximab, followed by IVIG [15]. The effectiveness of this protocol in significantly reducing pre-transplant HLA antibodies, however, has been variable. Despite this, some patients have successfully undergone transplantation with comparable post-transplant survival to those who did not receive desensitization therapy. These protocols can help expand the donor pool for sensitized patients by reducing the immunological barriers posed by pre-formed DSAs.

These findings further underscore that elevated cPRA values pose not only an immunologic barrier but also a significant logistical obstacle to timely lung transplantation. The reduced pool of compatible donors for sensitized patients contributes to prolonged wait times and increased pre-transplant mortality, emphasizing the need for adjustments in policy and allocation frameworks. Mangiola et al. [16] highlighted the role of anti-HLA antibodies as major impediments to transplantation, advocating for individualized immunologic risk assessment to facilitate donor acceptance and improve access for sensitized candidates. Similarly, Courtwright et al. [17] documented disparities in lung transplant access among sensitized populations—particularly among Black and Hispanic women—attributed in part to institutional variability in HLA antibody detection, interpretation, and management practices. These observations support the implementation of equity-driven reforms in lung allocation systems, including the integration of cPRA as a prioritization factor, as has been successfully adopted in renal and, more recently, heart transplantation programs [18,19]. Such approaches may help mitigate the disadvantages faced by sensitized candidates and promote more equitable access to transplantation.

This study has several limitations that should be acknowledged. First, it was conducted retrospectively at a single transplantation center, which may limit the generalizability of the findings to broader populations or different healthcare settings. Institutional practices, referral patterns, and regional donor availability may influence outcomes and may not be reflective of other transplant programs. Second, the analysis was limited to cPRA values obtained at the time of listing, without accounting for potential changes in sensitization status over time. Dynamic monitoring of cPRA and donor-specific antibody profiles could provide a more comprehensive understanding of immunologic risk and its temporal relationship with transplant outcomes. Additionally, the study did not incorporate less commonly used HLA loci or non-classical HLA forms, such as HLA-DP, HLA-E, HLA-F, and HLA-G, nor did it evaluate advanced immunologic assays (e.g., epitope mismatch algorithms or complement-binding capacity), which have shown potential in refining compatibility assessments. Future studies incorporating longitudinal data, multicenter cohorts, and expanded immunologic profiling are warranted to further elucidate the role of sensitization in lung transplantation.

## 5. Conclusions

This study highlights the significant impact of calculated panel-reactive antibody (cPRA) on lung transplantation waitlist dynamics at a lung transplantation center in Brazil. Elevated cPRA values were associated with prolonged waiting times and increased waitlist mortality, underscoring the dual role of sensitization as both an immunologic and logistical barrier to transplantation. Although the overall transplantation rate did not differ significantly across the cPRA strata, the marked reduction in transplant likelihood among highly sensitized patients aligns with prior literature on the topic and reinforces the need for policy revisions that integrate cPRA into allocation frameworks. The findings also emphasize the importance of targeted strategies—such as desensitization protocols, virtual crossmatching, and tailored delisting approaches—to improve access for sensitized candidates, particularly in genetically diverse and underserved populations. Despite its limitations, this study contributes with valuable insight into the challenges faced by sensitized patients and supports further investigation into individualized and equitable approaches to lung transplant candidacy and allocation.

## Figures and Tables

**Table 1:** The demographic distribution of patients included on the lung transplant waiting list.

	Total Subjects (n = 327)	Non-Sensitized Candidates—cPRA = 0% (n = 227)	Sensitized Candidates—cPRA > 0% (n = 100)	*p* Value
Median age (at inclusion on waitlist, years)	41 (10–67)	41 (10–65)	42 (11–67)	0.958
Female gender (%)	185 (56.6)	119 (52.4)	66 (66)	<0.01
Blood group (%)				<0.01
A	139 (42.5)	90 (39.6)	49 (49)	
B	32 (9.8)	24 (10.6)	9 (9)	
AB	11 (3.4)	8 (3.5)	3 (3)	
O	145 (44.3)	106 (46.3)	39 (39)	
Outcome				<0.01
Transplanted (%)	230 (70.4)	161 (71)	69 (69)	
Died whilst on transplant waitlist (%)	72 (22)	45 (19.7)	27 (27)	
Delisting due to clinical deterioration (%)	25 (7.6)	21 (9.2)	4 (4)	

**Table 2:** The demographic distribution of the transplanted patients.

	Transplanted Subjects (n = 230)
Median age (at transplant, years)	44 (10–66)
Female gender (%)	122 (53)
Blood group (%)	
A	108 (46.9)
B	26 (11.3)
AB	9 (4)
O	87 (37.8)
Lung disease group (%)	
Interstitial lung disease	98 (42.6)
Obstructive lung disease	37 (16.1)
Cystic fibrosis	54 (23.5)
Pulmonary vascular disease	8 (3.5)
Non-cystic fibrosis bronchiectasis	33 (14.3)
Median BMI	22.3 (17.1–32)
Median Class I cPRA	0 (0–75)
Median Class II cPRA	0 (0–99)
Median waitlist time (days)	551 (35–3151)

**Table 3:** Differences in cPRA values, waitlist time, and outcomes among the stratified groups according to the cPRA range.

	cPRA = 0%	cPRA 0.1–25%	cPRA 25.1–50%	cPRA 50.1–75%	cPRA > 75%	*p* Value
	n = 227	n = 54	n = 24	n = 14	n = 8	
Median Classes I/II cPRA (%)	0/0	6/0	26.5/0	56/26	12/81.5	<0.01
Transplanted patients (%)	161 (70.9)	37 (68.5)	19 (79.1)	10 (71.4)	3 (37.5)	0.277
Median waiting list time (days)	489 (35–1651)	565 (126–2001)	913 (252–2678)	742.5 (35–3151)	1288 (1213–1475)	<0.01
Delisted due to clinical deterioration (%)	21 (9.2)	3 (5.6)	1 (4.1)	0	0	0.722
Died whilst on transplant waitlist (%)	45 (19.8)	14 (25.9)	4 (16.6)	4 (28.5)	5 (62.5)	0.047

## Data Availability

The raw data supporting the conclusions of this article will be made available by the authors on request.

## Abbreviations

HLA	Human Leukocyte Antigen
DSAs	Donor-Specific Antibodies
cPRA	Calculated Panel-Reactive Antibody
BMI	Body Mass Index
CLAD	Chronic Lung Allograft Dysfunction
